# How far are volcanologists from volcanoes?

**DOI:** 10.1007/s00445-025-01849-6

**Published:** 2025-07-03

**Authors:** Gilles Seropian, Thomas J. Aubry, Jamie I. Farquharson, James Hickey

**Affiliations:** 1 Department of Earth and Environmental Sciences, University of Exeter, Penryn Campus, Penryn, TR10 9FE UK; 2 Present Address: Department of Earth Sciences, University of Oxford, Oxford, UK; 3 Institute for Research Administration, Niigata University, Ikarashi 2-8050, Nishi-ku, Niigata, 950-2181 Japan; 4 Research Institute for Natural Hazards and Disaster Recovery, Niigata University, Ikarashi 2-8050, Nishi-ku, Niigata, 950-2181 Japan

**Keywords:** Distance, Volcanologists, Volcanoes, Bibliometrics, Carbon footprint, Statistics

## Abstract

Volcanoes are not randomly located on Earth, neither are volcanologists. We explored the physical distance between volcanologists and volcanoes by considering two categories of volcanoes: all volcanoes with a confirmed eruption in the Holocene and volcanoes that erupted in the past 50 years (1974–2024). We computed the distance between these volcanoes and the affiliation addresses from volcanologists, defined here as authors having published in the main volcanology journals. We then investigated the dependence of this distance on further bibliometric parameters, such as the journal published in or the author position. Affiliations were extracted from articles published since 1980 in four of the main English-speaking volcanology-focused journals in the Scopus database. Around 27% of volcanologists are based within 100 km of a Holocene volcano, compared to 14% of the world population. More than 85% of volcanologists are within 1000 km of a Holocene volcano, but 48% need to travel over 1000 km to visit a volcano that erupted in the past 50 years. We tested whether distance to the nearest volcano correlates with author position. We observed that researchers working nearer volcanoes tend to lead articles with more co-authors, which is robustly supported by statistical tests. We also found that authors in further positions tend to be based nearer recently active volcanoes, though this correlation is less significant. Using keywords to identify each article’s studied volcano, we performed single volcano analysis for the 25 most frequently studied volcanoes in the dataset. We observed significant differences in the distance from authors to the target volcano. For instance, we obtained median author-volcano distances of 9 km for Campi Flegrei and 11,735 km for Merapi. Our analyses also permitted a very simplistic estimate of the carbon footprint from fieldwork travel, yielding CO$_{\varvec{2}}$ equivalent emissions of about 1 ton per travelling author, with an order of magnitude uncertainty. The database presented is very rich and could serve future efforts in science strategy, equality, diversity and inclusivity, outreach, and sustainability.

**Supplementary Information:**

The online version contains supplementary material available at 10.1007/s00445-025-01849-6.

## Introduction

Many volcanologists live and work in places quite far from any active volcano. For instance, the lead author is presently based in Cornwall, UK, over 1000 km away from the nearest currently erupting volcano (Reykjanes peninsula, at the time of writing). It is then very common to be faced with astonishment from the general public and questions regarding why such a peculiar location to study volcanoes. Based upon this recurring experience, we decided to investigate how common it is for a volcanologist to be based at great distances from volcanoes. Or in other words, how far are volcanologists from volcanoes? While seemingly a trivial question, there are several broader implications. Geography is fundamental in terms of both the generation and the consumption of scientific knowledge (Livingstone 2003), with science being shaped strongly by its spatial (i.e. geographical) context (Shapin 1998). The relative distance of publishing researchers to the object of their research is not only a case of physical proximity and logistics, but also involves matters of funding and availability of technology (e.g. Latour 1987), historical trends and context (e.g. Scarlett 2022), the existence of international and interdisciplinary collaborative networks (e.g. Wagner 2008; Adams 2012), and so on. In turn, these factors can strongly influence the research focus and methodology, funding opportunities, public engagement, and policy decisions. In a volcanological context, there may be important implications in terms of hazard awareness and mitigation (e.g. Barclay et al. 2022). Moreover, because volcanoes are spatially discrete (geographically constrained) features, their study may involve travel over great distances and collaboration between multiple parties, with attendant scholarly, societal, and climate implications. In this contribution, we seek first and foremost to identify whether the assumption that volcanologists—on average—work closer to active volcanoes, relative to the global population as a whole, holds true. To do so, we turn to bibliometric metadata analysis of journal articles published in a selection of international volcanology journals. Armed with these results, we then discuss patterns of publishing and authorship, some societal implications of studying far-flung volcanic systems, and take a step towards quantifying the carbon emissions associated with conducting fieldwork at active volcanoes.

Defining “volcanologists” constitutes a formidable challenge. Recent efforts by Kavanagh et al. (2022) and Lerner et al. (2023) have yielded a great overview of who and where volcanologists are; they have also raised issues related to equity, diversity, and inclusivity. Kavanagh et al. (2022) collated data from various sources, including memberships to international volcanology associations (e.g. the International Association of Volcanology and Chemistry of the Earth Interior, IAVCEI), bibliometric data (e.g. country of affiliation of lead-authors from volcanology journals), award winners, and anecdotal evidence from an online survey. Amongst their main findings, they showed that the majority of lead-authors are domiciled in Europe, North America, New Zealand, or Japan. Lerner et al. (2023) collected a very large amount of bibliometric metadata (e.g. title, affiliations, keywords) on a very wide range of published material (journal articles, books, conference proceedings...). They showed that, overall, 40% of the content about specific volcanoes does not include an author based in that volcano’s country.

Both studies generally used the country of affiliation as the basis for their geographical analysis (though Lerner et al. (2023) did distinguish mainland and island affiliations for volcanic islands), but did not consider true physical distance between research institutes and volcanoes. Calculating physical distance between a volcanologist and a volcano can add nuance for two main reasons. (1) Volcanoes do not care about human borders. This is particularly true for volcanoes located close to international borders, whose impacts can be significant on multiple countries (e.g. the 2011–2012 Cordón Caulle eruption in Chile mainly impacted neighbouring Argentina, Wilson et al. 2013; Forte et al. 2018). (2) The distance to a volcano can be very large within a single country, which is particularly true of very large countries like the USA or Russia (e.g. Semisopochnoi is >8000 km away from Miami, FL). Therefore, for questions relating to the inclusion of local researchers, country-level affiliations are not sufficient, and we must consider true distance. In this study, we test whether certain publishing parameters (author position, number of co-authors, and journal published in) are correlated with the physical distance to a volcano.

Volcanoes may be remote, even with respect to their local observatory (e.g. Semisopochnoi is monitored by the Alaska Volcano Observatory, based in Anchorage, $\sim$2100 km away). Studying volcanoes requires fieldwork, which generally involves long-haul flights, with a significant carbon footprint. Many articles have already pointed out that we, researchers, often suffer from “climate hypocrisy” (e.g. Høyer and Naess 2001; Caset et al. 2018; Higham and Font 2019). We tend to have better access and understanding of the climate crisis, but are amongst the most carbon emitting, in particular due to regular trips to conferences and the expected high level of mobility to pursue a career in academia (although with wide disparities within the community). With fieldwork being an essential component of volcanology, it is critical to assess its environmental impact in order to raise awareness and improve our practices as a community. Hence, the need for an accurate way to measure the actual distances travelled between a research institute and a field location. Here, we provide an initial, simplistic estimate of the carbon footprint from assumed fieldwork travel.

In this contribution, we used bibliometric data. We collected affiliation addresses from all authors who have published in the main volcanology-themed journals to compute the distance to the nearest volcano. We then extracted the name of the studied volcano from the articles keywords (where possible) and computed the distance to the studied volcano. The presented dataset offers many opportunities, with many parameters to investigate (journal published in, author position, country of affiliation...), and we provide examples of some of these possible analyses.

## Methods

We must tackle three problems: (1) How to define a volcanologist? (2) How to define a volcano? (3) And which “distance” metric is most relevant for our approach? No single answer exists to each of these three queries, and we detail below the different methods we employed in this study.

### Who are volcanologists?

To extract data for our volcanologists, we used bibliometric data, in the spirit of Kavanagh et al. (2022). We downloaded the metadata from all articles published since 1980 in four of the main English-language volcanology journals: the Bulletin of Volcanology (BV), the Journal of Volcanology and Geothermal Research (JVGR), the Journal of Applied Volcanology (JAV) and Volcanica. Data were downloaded from the Scopus database on 15 January 2024, using the +pybliometrics+ Python library (Rose and Kitchin 2019) and yielded 9816 articles. Limited metadata can be accessed for free from Scopus, but the full extent of the metadata requires a paid subscription to the database (acquired via the University of Exeter). Our approach is similar to Kavanagh et al. (2022), who considered articles from BV and JVGR only. It differs from Lerner et al. (2023), who, instead of focusing on certain journals, searched the whole database with the pattern “volcan*”, which resulted in almost 150,000 entries.

We are interested in where volcanologists are based, and therefore considered the list of affiliations for each article. For each affiliation, we attempted to geocode the pattern “*Affiliation Name, City, Country*” (i.e. obtain coordinates given a name), using the Nominatim geocoding tool from OpenStreetMap. Our bibliometric database included 2871 affiliations, 1353 of which (47%) were successfully geocoded. For the remaining 1518 affiliations, we geocoded “*City, Country*” only, which generally returned the city centre coordinates. We estimate that for most institutes, this would represent a $<25$ km error in location. Eleven affiliations (0.4%) failed this second step and were geocoded manually.

We noted a few limitations with this method. Firstly, some institutions have branches in different locations, but the Scopus database only lists the main headquarters. For instance, the University of Exeter is listed in the city of Exeter, but the volcanology group in the Department of Earth and Environmental Sciences is physically based at the Penryn Campus in Penryn, Cornwall, 125 km further west. Whilst the error is not significant in this case, there may be more dramatic cases: the Observatoire Volcanologique du Piton de la Fournaise is based in La Réunion, but listed as part of the Institut de Physique du Globe de Paris, over 9000 km away. We manually fixed these two instances, but we acknowledge that there are likely other such issues that we are unaware of. Regardless, we believe that most affiliations will be correctly located to within a few tens of kilometres. A second potential issue arises when multiple cities with the same name exist in the same country. For instance, “Saint-Denis, France” could refer to a city 10 km northeast of Paris or to the capital city of La Réunion, 9375 km away. This issue is only valid for affiliations that could not be geocoded in the first step (i.e. including the institution name).

Following geocoding, we created two databases. The first database is a list of all 2871 affiliations, where each affiliation is represented once, regardless of how many times they have published, and further referred to as “Affiliation database”. The second database is a list of all affiliations weighted by the number of times an author has published under this affiliation, resulting in 43,634 entries, referred to as “Author database”. Authors with multiple affiliations ($n=3791$) are counted once for each affiliation.

Our definition of a volcanologist is quite restrictive, as it requires publication in a specific subset of English-speaking peer-reviewed journals. The issue of the restrictive volcanologist definition is partially remedied with the Affiliation database, and we discuss this limitation in more detail in “Limitations of the dataset”.

### Which volcanoes?

**Fig. 1:**
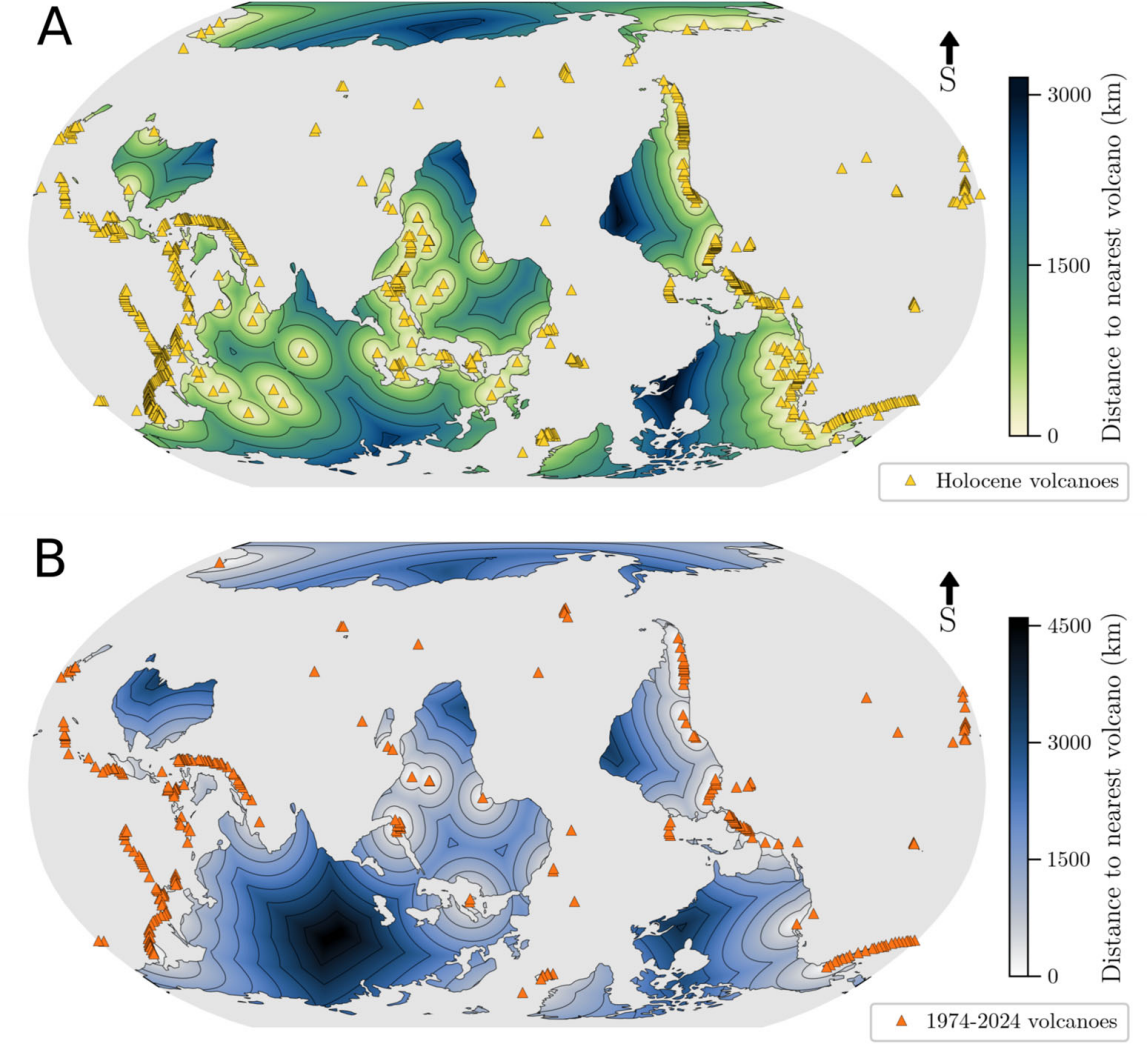
Heatmaps of the world where colour represents distance to the nearest volcano for **A** the Holocene and **B** the last 50 years volcano lists. Contour lines are plotted every 500 km

We used two different lists of volcanoes, based on the Global Volcanism Program (GVP) database (Venzke 2023). A generally accepted definition for an active volcano is one that erupted in the Holocene (Szakács 1994). Therefore, we first considered the GVP list of volcanoes with at least one confirmed eruption during the Holocene. We restricted our attention to subaerial volcanoes and obtained a total of 847 volcanoes (Fig. 1A). We note, however, that filtering all “Undersea Features” from the GVP database does not remove all underwater volcanoes. Volcanic centres like the North Gorda Sea Segment in the NE Pacific or multiple unnamed vents around Tonga remain in the list. Including volcanoes with unconfirmed Holocene eruptions yields almost identical results and is thus not considered further.

Whilst providing a good basis for our study, the Holocene-eruptions volcano list does include volcanoes that have not erupted for thousands of years (an issue already raised and extensively discussed by Szakács 1994). The motivation for our study was the general audience perception of how far is the nearest volcano. Thus, we also focused on the volcanoes that have erupted in the past 50 years only, i.e. in the period 1974–2024, which yielded a total of 293 volcanoes (Fig. 1B).

As noted by Lerner et al. (2023), the GVP database comes with its idiosyncrasies. For instance, Lerner et al. (2023) identified six regularly studied volcanoes that are not identified as such in the database, but rather grouped within larger volcanic centres. These are (with their respective volcanic centres): Surtsey (Vestmannaeyjar), Tarumae (Shikotsu), Ngāuruhoe (Tongariro), and North Sister, Middle Sister, and South Sister (Three Sisters). Given the proximity of these volcanoes to their “parent” volcanic centres ($<25$ km), the grouping does not add significant uncertainty to our calculations, and we did not manually split them.

We also noted that, despite hosting one of the few lava lakes in the world, Mount Erebus was absent from our initial list of volcanoes with eruptions from the last 50 years. Indeed, the “Last Known Eruption” of Mount Erebus in the GVP database is listed as having started in 1972 (i.e. 52 years ago), and still ongoing. We manually added Mount Erebus to our list. The GVP database lists five other volcanoes with ongoing eruptions that started before 1974 (Dukono, Erta Ale, Santa Maria, Stromboli, and Yasur), but these were all correctly picked up by our filtering algorithm.

### How far?

Our spatial analysis was performed using the haversine formula to compute distance *d* along a great circle between two points on Earth with latitudes $\varphi _{1,2}$ and longitudes $\lambda _{1,2}$:

$$\begin{aligned} d = 2R\arcsin \left[ \sqrt{\sin ^2(\frac{\varphi _1-\varphi _2}{2}) + \cos \varphi _1\cos \varphi _2\sin ^2(\frac{\lambda _1-\lambda _2}{2})}\right] , \end{aligned}$$

where $R=6371$ km is Earth’s radius (Chambat and Valette 2001). The haversine formula can result in errors up to 0.33% because Earth is not a perfect sphere (e.g. Mahmoud and Akkari 2016). Most distances considered in this study are <5000 km, leading to maximum errors <17 km. Given our purpose, we consider this satisfactory and did not attempt to reduce this uncertainty further.

For each affiliation, the nearest volcano was found in both volcano lists using the Ball Tree nearest neighbour search algorithm from the +scikit-learn+ library (Pedregosa et al. 2011). We also explored alternative metrics, focusing on the “density” of volcanoes around a given location: we calculated the distance to the 10^th^ nearest volcano and the number of volcanoes in a 1000 km radius around each affiliation.

**Fig. 2:**
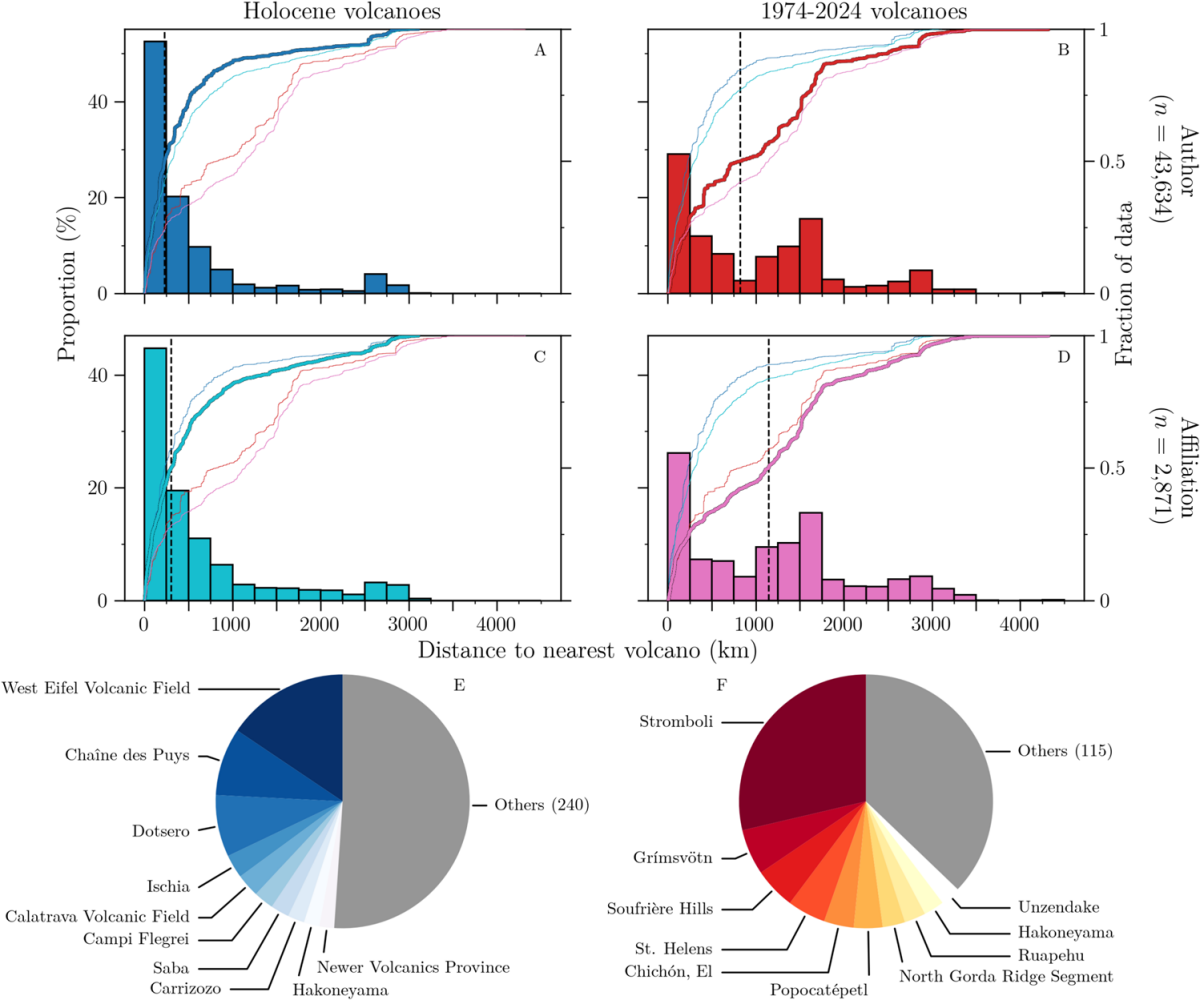
**A**–**D** Frequency distributions (as percentage) of the distance from volcanologists to the nearest volcano, in 250 km bins. The thick, matching-colour, line is the corresponding cumulative curve, with *y*-scale on the right hand side. Other cumulative curves are plotted as thin coloured lines for comparison on all panels. The dashed vertical black lines indicate the medians. **E**, **F** The ten most frequent nearest volcanoes are displayed as pie charts for the Affiliation database, combined with **E** the Holocene and **F** the 1974–2024 volcano lists

## Results

### Distance to nearest volcano

Our analysis combines two volcano lists (Holocene, and 1974–2024) with two databases of volcanologist locations (Affiliation and Author), thus yielding four different distributions (Fig. 2A–D). In all four distributions, the main mode is in the range 0-250 km. We emphasize here that this is only the distance to the nearest volcano, which is not necessarily the volcano studied by researchers. The distance to the studied volcano is discussed in “Distance to studied volcanoes”.

The two main observations are that: (1) the choice of volcano list (Holocene or 1974–2024) exerts a major influence on the resulting distributions, but (2) the distributions are not very sensitive to the choice of volcanologists database (Author or Affiliation). These two observations are particularly visible in the cumulative plots, where the two curves from the same volcano list follow each other closely (Fig. 2A–D).

When considering Holocene volcanoes (Fig. 2A and C), the distributions are heavily skewed towards short distances, with >40% of the volcanologists based within 250 km of a volcano in both cases, and medians of 229 and 304 km, for the Author and Affiliation databases, respectively. This initial large peak is followed by a rapid decrease, before a small but noticeable secondary peak occurs in the range 2500–3000 km. For 1974–2024 volcanoes (Fig. 2B and D), the two distributions are bimodal and the medians increase by a factor >3.5, to 825 and 1145 km, respectively. The dominant peak remains within 0–250 km, though it is much weaker (<30%), and a second, strong mode ($\sim$18%) appears around 1500 km.

For each distribution, we can obtain the most frequent “nearest volcanoes”. For both volcano lists combined with the Affiliation database, the “top 10 nearest volcanoes” are dominated by European and North American volcanic centres (9 and 7 for Holocene and 1974–2024, respectively, Figs. 2E and F). The same analysis was carried out with the Author database instead, with almost identical results.

### Bibliometric parameters

We can interrogate the Scopus database further to explore how distance to the nearest volcano varies with different bibliometric parameters. In the following section, we only consider the Author database as the choice of volcanologist database only has a limited influence on the resulting distributions (Fig. 2). We do, however, continue to use both volcano lists (Holocene and 1974–2024).

#### By publication

**Fig. 3:**
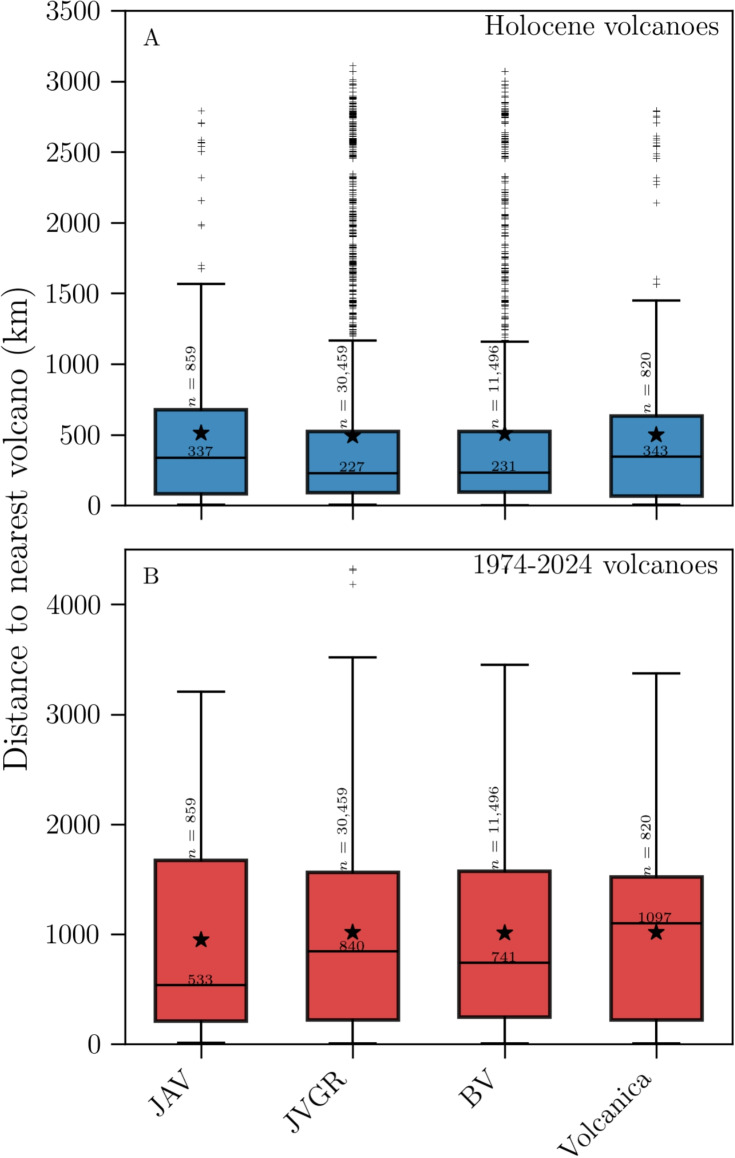
Distributions of the distance between volcanologists and their nearest volcano, grouped by journal, for the Author database with **A** the Holocene and **B** the 1974–2024 volcano list. Boxes are drawn from the first to the third quartile, with the median shown as a black line and the mean as a black star. Whiskers extend to the last point falling within 1.5 interquartile range (i.e. whiskers do not represent uncertainty); all other points are plotted as crosses

The distance distributions (Fig. 2A, B) can be further divided according to which journal the authors published in (Fig. 3). Due to different durations of existence, the number of samples varies greatly between journals, with >30,000 for JVGR and <1000 for JAV and Volcanica. Nonetheless, the distributions are relatively similar for all journals when using the Holocene volcano list (all medians within 220–350 km, Fig. 3A). With the 1974–2024 list, the distributions still show very similar ranges and averages, but the medians vary more significantly (Fig. 3B). JAV features the smallest median at 533 km, followed by BV and JVGR at 741 and 840 km, respectively. The median for Volcanica lies at 1097 km, over two times more than JAV.

**Fig. 4:**
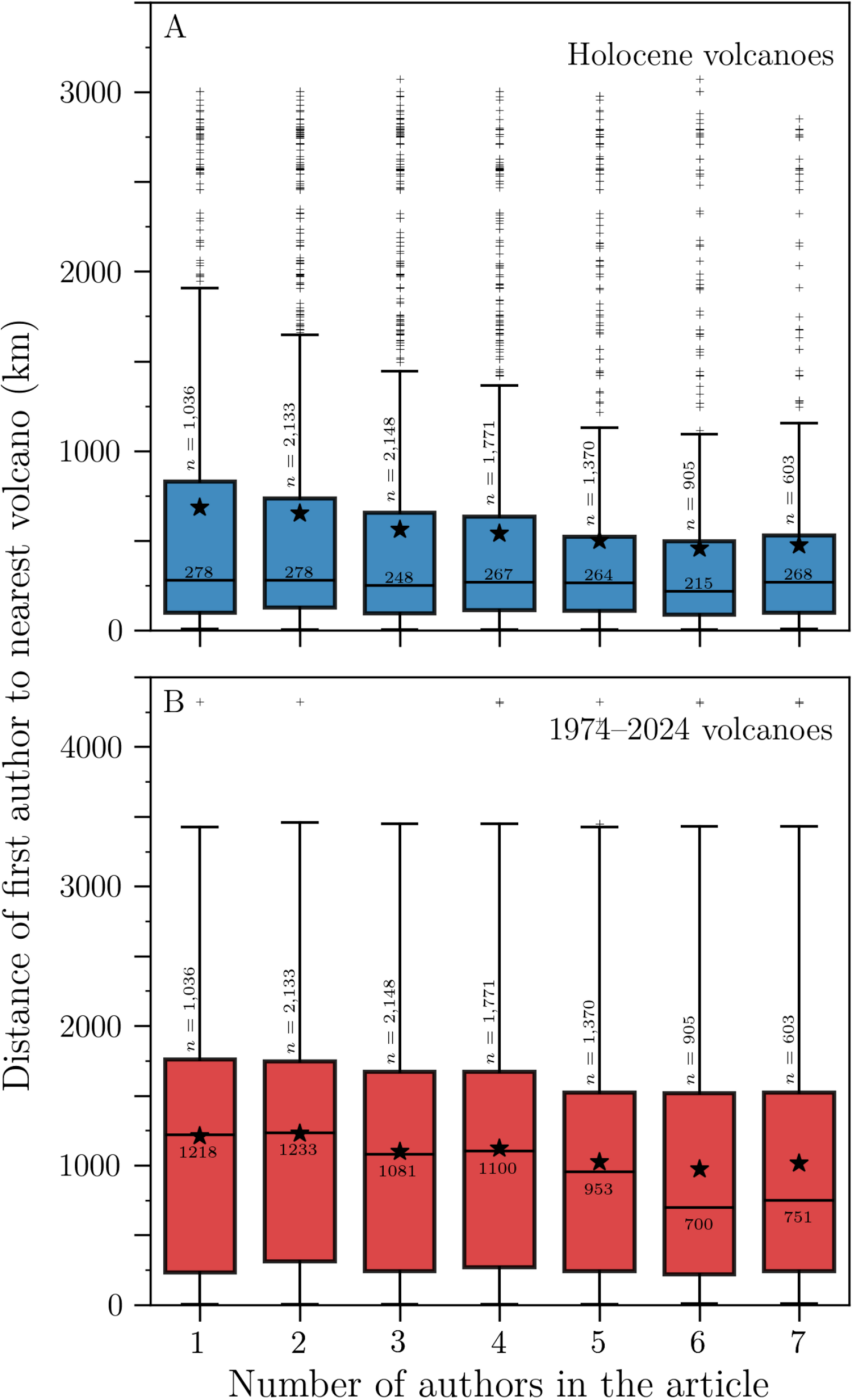
Distributions of the distance between first authors and their nearest volcano, as a function of the number of authors in the article, for the Author database with **A** the Holocene and **B** the 1974–2024 volcano list. Boxplot parameters and symbols are the same as in Fig. 3. Kendall’s $\tau _B$ coefficients for the medians are **A** $-$0.39 and **B** $-$0.71, with *p*-values of 0.22 and 0.03, respectively

#### By author position

In a similar fashion, we analyzed the distance distributions, as a function of author position in the author list. The total number of authors in a given article varies between 1 and 42, with 97% of articles having 1–10 authors. We first investigated whether the distance to the nearest volcano of a given author position is correlated to the number of authors in the article. We restricted our analysis to the first seven author positions, to ensure sufficient sample size for statistical analysis. For instance, the distances to the nearest volcano for first authors seem to be negatively correlated to the number of authors (Fig. 4). We computed Kendall’s rank correlation coefficient $\tau _B$ for the set of medians. $\tau _B \in [-1, 1]$ provides an estimate of the strength of a monotonic correlation between two variables. We obtained −0.39 and −0.71, for the Holocene and 1974–2024 lists respectively, with *p*-values of 0.22 and 0.03, confirming moderate to strong negative correlations between the median first author distance to the nearest volcano and the number of authors in the article. Similar analyses can be performed for all author positions, all resulting in negative correlations with variable significance (Table 1).

**Table 1:** Kendall’s $\tau _B$ values for the medians of the distance distributions of author positions 1–4 as a function of the number of authors

-	Holocene		1974–2024	
Author position	$\tau _B$	*p*-value	$\tau _B$	*p*-value
1	$-$0.39	0.22	$-$0.71	0.03
2	$-$0.33	0.47	$-$0.97	0.007
3	$-$1	0.02	$-$0.80	0.08
4	$-$0.33	0.75	$-$0.55	0.27

**Fig. 5:**
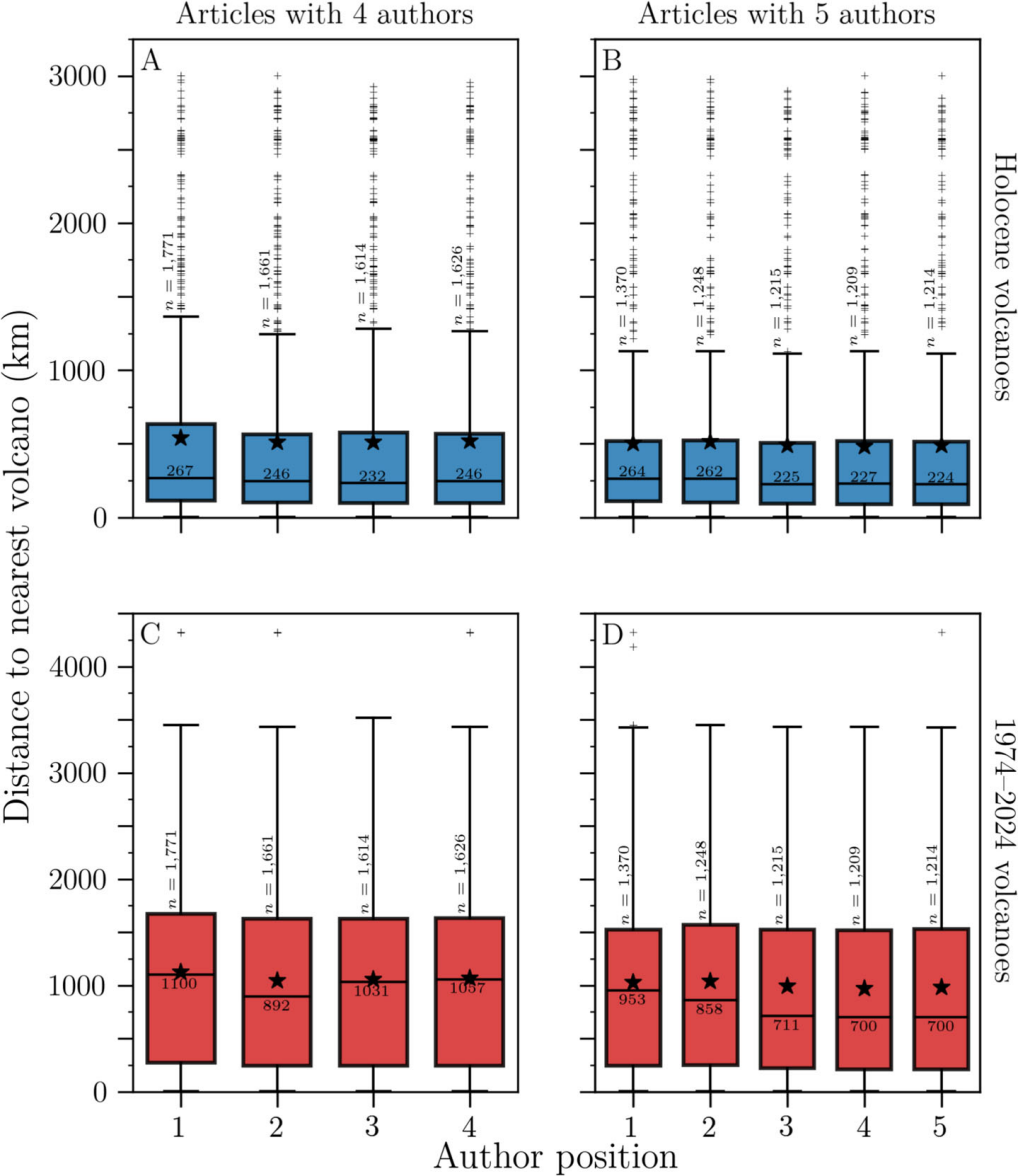
Distributions of the distance between volcanologists and their nearest volcano, as a function of author position, for the Author database. The first column (**A**, **C**) focuses on articles with a total of 4 authors, whereas the second column (**B**, **D**) considers articles with 5 total authors. The first row (**A**, **B**) uses the Holocene volcano list, and the second row (**C**, **D**) uses the 1974–2024 volcano list. Boxplot parameters and symbols are the same as in Fig. 3

The distance to the nearest volcano for a given author position is thus negatively correlated with the number of authors in the article. Hence, in order to explore whether the distance to the nearest volcano depends on author position, we compared articles with the same number of authors. The distributions for articles with four and five authors are shown as boxplots in Fig. 5. We calculated Kendall’s $\tau _B$ values for the set of medians and performed Kruskal-Wallis significance *H* values on the whole distributions (Table 2). Kruskal-Wallis test compares the whole distributions (rather than just the medians for $\tau _B$) and indicates whether they come from the same parent distribution or at least one of them differs. In general, and apart from the case of articles with 4 authors, using the 1974–2024 volcano list (Fig. 5C), there is a moderate to strong negative correlation between distance to the nearest volcano and author position, as shown by $\tau _B$ values in the range $-$0.58 to $-$0.95. The statistical significance of these trends varies with *p*-values <0.1 for all but three cases (Table 2). The Kruskal-Wallis *H* statistics are less striking, with only three cases featuring *p*-values <0.1. Overall, our results suggest that authors in further positions are generally closer to volcanoes, though the magnitude of this trend is sensitive to the choice of dataset.

**Table 2:** Kendall’s $\tau _B$ values (for the set of medians) and Kruskal-Wallis *H* statistics (whole distributions) for the distance to the nearest volcano as a function of author position, for articles with 4–7 authors

Number of	Holocene				1974–2024			
authors in article	$\tau _B$	*p*-value	*H*	*p*-value	$\tau _B$	*p*-value	*H*	*p*-value
4	$-$0.67	0.33	3.57	0.31	0	1	8.05	0.04
5	$-$0.80	0.08	8.65	0.07	$-$0.95	0.02	9.44	0.05
6	$-$0.60	0.14	2.90	0.71	$-$0.89	0.02	6.30	0.30
7	$-$0.71	0.03	7.68	0.26	$-$0.58	0.08	7.35	0.29

### Other distance metrics

**Fig. 6:**
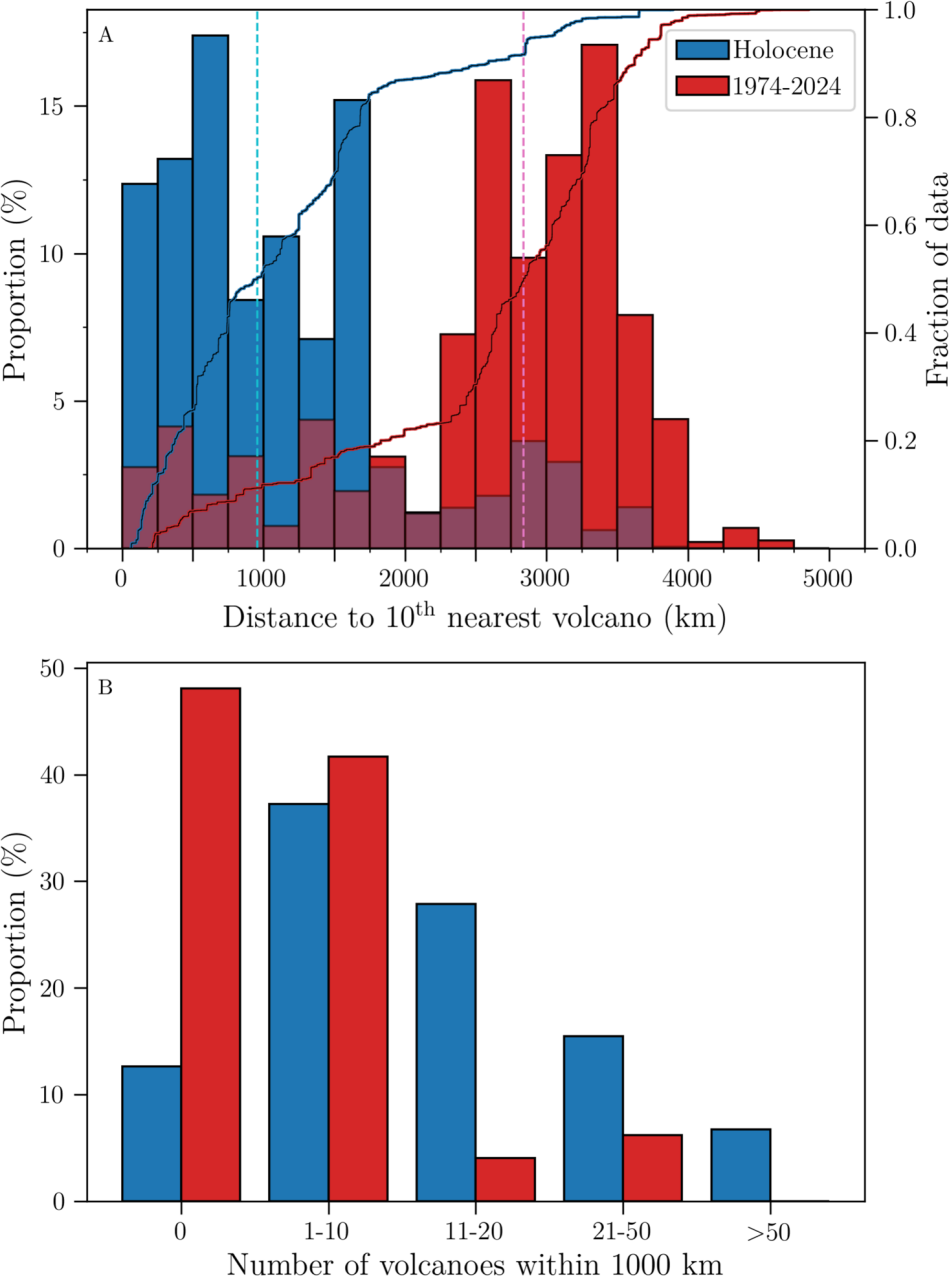
Examples of other possible metrics used to quantify how far volcanologists are from volcanoes, with the Author database. **A** Distributions of distances to the 10th nearest volcano, with corresponding cumulative curves. The dark purple colour represents the overlap of the two distributions. The dashed light blue and pink lines represent the Holocene and 1974–2024 medians, with respective values of 954 and 2838 km. **B** Number of volcanoes within 1000 km of a volcanologist’s location. *X*-scale is not linear. Bar colours correspond to those in **A**

The number of potential metrics that can be used is vast. So far, we have focused on direct distance (haversine formula) between a volcanologist’s affiliation and the nearest volcano. Alternative metrics can be used to quantify how “surrounded” by volcanoes, a given location is (Fig. 6).

We calculated the distance to the 10th nearest volcano (Fig. 6A); the difference between the Holocene and 1974–2024 data is very marked. The Holocene distribution features a very broad peak between 0 and 1750 km, followed by a sudden decrease, and a much smaller secondary mode around 3000 km. The 1974–2024 data feature an initial noisy distribution but relatively constant around 2.5%, between 0 and 2250 km, before a main, broad, bell-shaped peak in the range 2250–4000 km, and a final very minor decaying tail, with a maximum value of 4856 km.

Another approach to measure the “density” of volcanoes around a given point is to compute how many volcanoes are present in a given radius, for example 1000 km (Fig. 6B). In the Holocene case, the majority of volcanologists have multiple volcanoes within 1000 km of their base location, with 7% having >50 volcanoes in that range. Regarding volcanoes that erupted in the past 50 years, almost half of the volcanologists (48%) are located >1000 km from such a volcano, but 42% have 1–10 such volcanoes within 1000 km of their office.

## Discussion

### Are volcanologists near volcanoes?

Our results indicate that most volcanologists work within a few hundred kilometres of a volcano, regardless of the definitions used. The Author distributions show medians of 229 and 825 km (Holocene and 1974–2024 lists, respectively). Our results can be compared to the global medians of all land on Earth of 1043 and 1579 km. These numbers should however be nuanced because most land on Earth is uninhabited. In particular, many of the regions furthest away from volcanoes are also regions with very low population densities, such as central Quebec, central Russia, or Antarctica (Fig. 1). An interesting future effort could be to combine the data presented in Fig. 1 with a global population distribution in order to produce a weighted map.

Nevertheless, recent estimates suggest that about 14% of the world population lives within 100 km of a Holocene volcano (Freire et al. 2019). We showed that >40% of volcanologists are based within 250 km of a Holocene volcano (Fig. 2A and C). Refining our analysis to 100 km bins, we find values of 26 and 28% for the Author and Affiliation databases, respectively, within 100 km of a Holocene volcano, suggesting that volcanologists are indeed more likely to be located close to a volcano than any randomly chosen individual. We do, however, emphasize that the uncertainties on our distance estimates are of order 20–50 km, due to uncertainties in both volcano coordinates (volcanic centres can be very extensive) and in geocoding results. An exciting future prospect is to turn Fig. 1 into an interactive map, where the user could navigate the world, and toggle different volcano filters to find out what is the nearest volcano. Such a tool would be particularly useful for outreach activities. An initial version is being developed online where users can search for the nearest volcano from a given institution, or reverse search the nearest research institutions from a given volcano (see Supplementary Material)

### Bibliometric implications

It is difficult to draw significant conclusions about how the distance to the nearest volcano varies for authors in different journals due to the extreme differences in sample size (Fig. 3). Nonetheless, there is a large difference in the medians of the Journal of Applied Volcanology (533 km) and Volcanica (1097 km) distributions, when considering the 1974–2024 volcano list (Fig. 3B). The sample size, overall range, and mean are otherwise similar. Performing a Kolmogorov-Smirnov test on these two distributions yields a *p*-value $\ll$1. The difference in distributions could be a reflection of the journals’ scopes. JAV focuses on applied research and societal impacts, which seems more likely to be conducted by scientists living near recently active volcanoes. In contrast, Volcanica promotes all aspects of volcanology and thus reaches a much wider range of researchers.

Our results indicate that first authors tend to be located nearer volcanoes (especially recently active ones) for larger collaborations, whereas articles with fewer authors are led by scientists further away. There are likely several factors explaining this trend. One might relate to the type of work performed, with, for instance, fieldwork generally requiring more people than numerical modelling or remote sensing. These trends might also reflect different research practices and co-authorship cultures. For example, many research articles produced at universities far away from a volcano may include a student with one or two co-supervisors, whereas articles from observatories may include a whole team.

An interesting future step would be to explore how the distance distributions evolve with time. The influence of various parameters such as new technology (e.g. remote sensing) or new best practice recommendations (e.g. Engagement Protocols for International Collaboration, IAVCEI-INVOLC International Network 2024) could then be evaluated. One difficulty would be to define the list of volcanoes that erupted in the last 50 years—each year would have a slightly different list. Similarly, the list of articles considered would need to vary with time, but the publication date in the Scopus database is not always very accurate, in particular for older entries. Whether this uncertainty would constitute a major limitation remains to be tested.

Another avenue for future work is to use keywords to identify the type of work performed in an article. One goal would be to differentiate methods requiring direct access to a volcano (e.g. fieldwork) from methods that can be employed remotely (e.g. satellite monitoring, numerical modelling) and test whether the resulting distance distributions differ. Lerner et al. (2023) performed a similar analysis, focusing on the inclusivity of local researchers, but they found no significant systematic difference with remote sensing articles.

### Limitations of the dataset

Our results give insight into the geographical distribution of volcanologists in the world, and how it relates to the distributions of volcanoes. Our geographical analysis is based on bibliometric data from four of the main English-speaking peer-reviewed journals focusing on volcanology. Our choice of dataset is restrictive, and the resulting data may not be representative of the actual distribution of volcanologists.

Most volcanologists will publish in many journals other than the four selected ones. However, the four journals chosen here focus on volcanology explicitly, which facilitated the initial data selection. Other broader scope journals (e.g. Journal of Geophysical Research) will contain a majority of non-volcano related contributions, requiring more involved filtering (Lerner et al. 2023). The chosen journals are also English-speaking only (though BV and Volcanica offer the option to publish abstracts in different languages). Although the majority of volcanoes are not located in English-speaking countries, most peer-reviewed publications are produced in English (e.g. Curry and Lillis 2017). The most notable exception for volcanology is the Bulletin of the Volcanological Society of Japan which is published in Japanese. Omitting this journal may have led to under-representation of Japanese-speaking volcanologists in our dataset.

We only considered peer-reviewed publications, but access to the publication process is not even across the world (e.g. due to high costs or language barriers, North et al. 2020; Ramírez-Castañeda 2020). Additionally, volcanologists produce a significant amount of non-peer-reviewed material, such as monitoring reports or press releases (e.g. Peltier et al. 2022). Therefore, our dataset is biased towards volcanologists with the ability to produce many peer-reviewed publications in English. The Affiliation database helps to reduce some of this bias because affiliations are only counted once, regardless of the number of publications. The requirement then becomes that at least one researcher has co-authored an article in the four considered journals, since 1980. While it does still require publication in an English-speaking journal, this criterion is fulfilled by many research institutes and observatories, including in non-English-speaking, low-income regions. A direct consequence however, is that many institutions that do not primarily do volcano research appear with equal weight in this database. In particular, institutions or companies that have participated in a small number of ad hoc collaborations on volcano research are disproportionately represented in the database, with regards to how many volcanologists are actually located there. Both databases thus suffer from different biases, but the resulting distributions are very similar (Fig. 2).

**Fig. 7:**
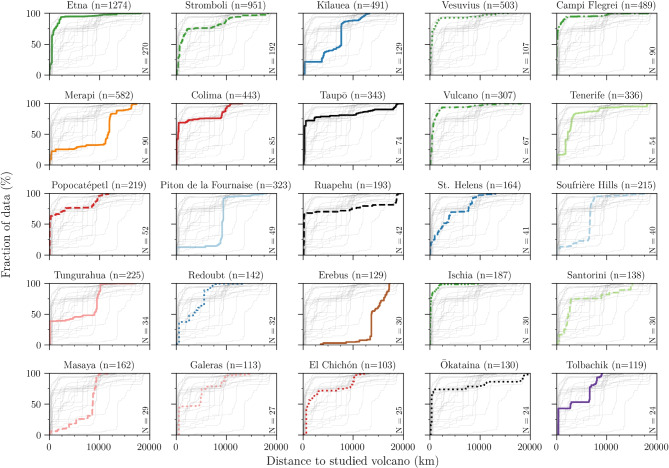
Cumulative curves of the frequency distributions of the distance between authors and the 25 most frequently studied volcanoes (by basic keyword search). Curves are colour-coded by broad regions (see main text). *n* is the number of authors and *N* is the number of articles considered for this volcano

### Distance to studied volcanoes

The results presented here indicate how far the nearest volcano is to a given author, but do not specify whether the author actually studied that volcano, one much further away, or no specific volcano at all. To illustrate this, suppose two researchers study Ubinas volcano in southern Peru, and one is based in Lima, Peru, and the other one in Reykjavik, Iceland. According to our analysis, the Lima researcher is 632 and 697 km away from the nearest volcanoes (Andahua-Orcopampa and Sabancaya, for the Holocene and 1974–2024 lists, respectively), whereas the Reykjavik researcher is 25 km away from Brennisteinsfjöll (Holocene) and 32 km from Fagradalsfjall (1974–2024). Researchers are not entirely defined by their current affiliation however. It is possible that the Reykjavik researcher is a Peruvian citizen, or has spent extensive time working in Peru in a previous position. All these subtleties significantly increase the difficulty of quantifying the “distance” between volcanologists and the volcanoes they study.

Nevertheless, we can interrogate our database, albeit with a slightly modified method. Instead of measuring distance to the nearest volcano, we compute the distance to the studied volcano. In the previous example, this would yield 817 and 9870 km for the distances from Ubinas to Lima and Reykjavik, respectively. This is in line with the method used by Lerner et al. (2023).

The main difficulty is to determine the volcano studied. The Scopus database includes the list of keywords for each article. We searched for volcano names in these keywords, and then computed the distance between the affiliations of each author to that specific volcano, using the coordinates obtained earlier from geocoding. As an example, we provide the results of such an analysis for the 25 most frequent volcanoes in keywords (Fig. 7).

The keyword search performed suffers from several weaknesses, many of which have already been identified by Lerner et al. (2023). They identified 60 volcanoes with multiple commonly used names (e.g. Changbaishan is also referred to as Changbai, Paektu, Baekdu, or Tianchi, Lerner et al. 2023). Conversely, some volcanoes have the same name (e.g. Flores in Guatemala and Flores in the Azores, Portugal, Lerner et al. 2023). We already mentioned six volcanoes that are grouped within a larger volcanic centre and thus absent from our volcano lists (e.g. Ngāuruhoe is considered part of Tongariro, see “Methods” and Lerner et al. 2023). Additionally, some of the volcano names in the GVP list are accurate but differ slightly from the more commonly used names (e.g. Puyehue-Cordón Caulle usually only appears as Cordón Caulle). Some volcanoes may also be referred to via specific eruptive vents or eruptions (e.g. Cumbre Vieja, Tajogaite for La Palma, or Puy de Dôme for Chaîne des Puys). Some volcanoes have names with alternative meanings that may appear in other, unrelated keywords (e.g. Late, Tonga). All these issues are not problematic for our purpose, as we merely provide an example of how the analysis can be performed for some of the most studied volcanoes. However, these factors must be taken into account for detailed analysis of specific volcanoes.

Logically, distances to the studied volcanoes (Fig. 7) are much greater than those to the nearest volcanoes (Fig. 2). The distributions medians for the 25 “most studied volcanoes” span a very large range, with the two lowest values being 9 and 68 km (Campi Flegrei and Popocatépetl, respectively), and the two highest values of 11,735 and 13,598 km for Merapi and Mt Erebus. For comparison, the maximum possible distance between two points on Earth (i.e. antipodes) is about 20,000 km. Visualizing the resulting distributions as cumulative curves helps identify different trends. The curves in Fig. 7 are also colour-coded by broad geographic region, which we detail below.

Italy (6 volcanoes) is in dark green, and these curves consistently show a very fast rise, with >80% of authors studying Italian volcanoes within 1000 km. The rest of Europe (2) is in light green (Spain, 1; Greece, 1). The two curves follow each other closely with an initial slow rise, followed by a sharp increase between 2000 and 3000 km, consistent with the location of these volcanoes being a few thousand kilometres away from the main European research centres (e.g. Tenerife is 1790 and 2240 km away from Madrid and Barcelona, respectively).

UK (1) and France (1) volcanoes are grouped together in light blue because these volcanoes are located in distant overseas territories. Their cumulative curves are very similar, with an initial rise, from researchers based in local observatories, followed by a plateau until very large, sudden increases at $\sim$6500 and 9200 km, respectively. These two distances match represent the distances between these volcanoes and their respective mainlands (e.g., Soufrière Hills–London is 6640 km and Piton de la Fournaise–Paris is 9410 km).

USA volcanoes (3) are in dark blue and show steady trends, reaching 80% at $\sim$7700 km, consistent with these volcanoes being remote within the USA, but studied by a variety of researchers across the country. Note the very large step at 7615 km for Kīlauea, which corresponds to the distance to Reston, VA, where the main USGS headquarters are located. Russia (1) is in dark purple, and features a staircase cumulative plot. The first and third large steps (40% at $\sim$330 and 30% at 6600 km) represent the distances from Tolbachik to Petropavlovsk-Kamchatsky (330 km) and Moscow (6570 km), respectively. The second step at 2700 km (10%) comes from the distance to Japanese research centres (e.g. Tolbachik–Tokyo is 2730 km).

Volcanoes in Mexico (dark red, 3) and New Zealand (black, 3) show similar trends with 60–70% of the authors within 1000 km, followed by steady, prolonged increases (in particular for New Zealand, which features the longest author-volcano distance at 19,830 km for Madrid–Taupō). These trends indicate that the volcanoes analyzed for Mexico and New Zealand are mostly studied by local volcanologists, with many international collaborations.

Colombia (1), Ecuador (1), and Nicaragua (1) are displayed in pink, but show varied trends. The trend for Tungurahua features two very large steps, a close one (40%) at 140 km, from the main observatory in Quito, and another one (50%) at 9500 km, corresponding to the distance to Europe. Galeras also displays an initial large step at 530 km (distance to Bogotá), followed by a more spread out step between 4000 and 6000 km (distance to various research centres in North America), and a last step between 9000 and 10,000 km (distance to Europe). Therefore, this suggests that local volcanologists represent about 40% of the authors publishing on these volcanoes, and 60% of the authors come from international collaborations. The case of Masaya is more dramatic, with the first 30% of authors evenly spread out over 8500 km, followed by a major, sudden increase to nearly 100% between 8500 and 10,000 km. These distances represent the path to European cities, demonstrating that the majority of researchers publishing on Masaya, in the considered journals, are from Europe. As mentioned earlier, the type of data chosen here is biased because it only includes English-speaking journals, as well as volcanologists with the resources to publish in these journals (Volcanica withstanding, due to its Diamond Open Access nature).

Indonesia (1) is depicted in orange, with Merapi the sixth most frequently studied volcano in our database. The first 20% occur within 500 km, from Java-based researchers. The cumulative curve then rises, steadily but very slowly, until $\sim$11,000 km, at which point it jumps up very fast, corresponding to European authors. The 50% mark (median) is 11,735 km. As for the case of Masaya, this suggests that most volcanologists publishing on Merapi are based in Europe, though the same language limitation applies here. This result matches the outcomes from a literature review on natural hazards in Indonesia, which showed that only half of the publications involved Indonesian researchers (Djalante 2018).

Finally, Mt Erebus is shown in brown, and provides an extreme end-member example because it is located in Antartica. The closest authors are 3520 km away, in Dunedin, New Zealand, and a dominant increase occurs at 13,600 km, from researchers based in North America.

### Fieldwork’s carbon footprint

Our volcano specific analysis may also be used to broadly quantify the carbon footprint from travels to studied volcanoes. We have computed distances to some of the most studied volcanoes (Fig. 7). These distances can be converted to emissions of CO$_2$ equivalent (CO$_2$e). The conversion factor depends on a series of parameters such as the means of transport (e.g. aeroplane, car), the distance travelled (e.g. short-haul vs long-haul flight), the number of travellers (e.g. single-passenger vs car-pooling), or how modern the vehicle is. A number of methodologies exist to choose an appropriate conversion factor, each with varying assumptions and uncertainties (e.g. Jardine 2009). Given the simplistic nature of our estimate, we used a range of values from 50 to 300 g/km of CO$_2$e, encompassing the most common values from the French (ADEME 2024) and British (DESNZ 2024) official conversion factor databases (Table 3).

**Table 3:** Examples of distance to CO$_2$e conversion factors (g/km) for various means of transportations in the British (DESNZ 2024) and French (ADEME 2024) databases

Transport	DESNZ 2024	ADEME 2024
Combustion engine car	140–207	105–259
Short-haul flight	183–272	187–527
Long-haul flight	135–200	152–178
Long-distance train	4–35	3–129
Ferry	19–129	229–523

**Fig. 8:**
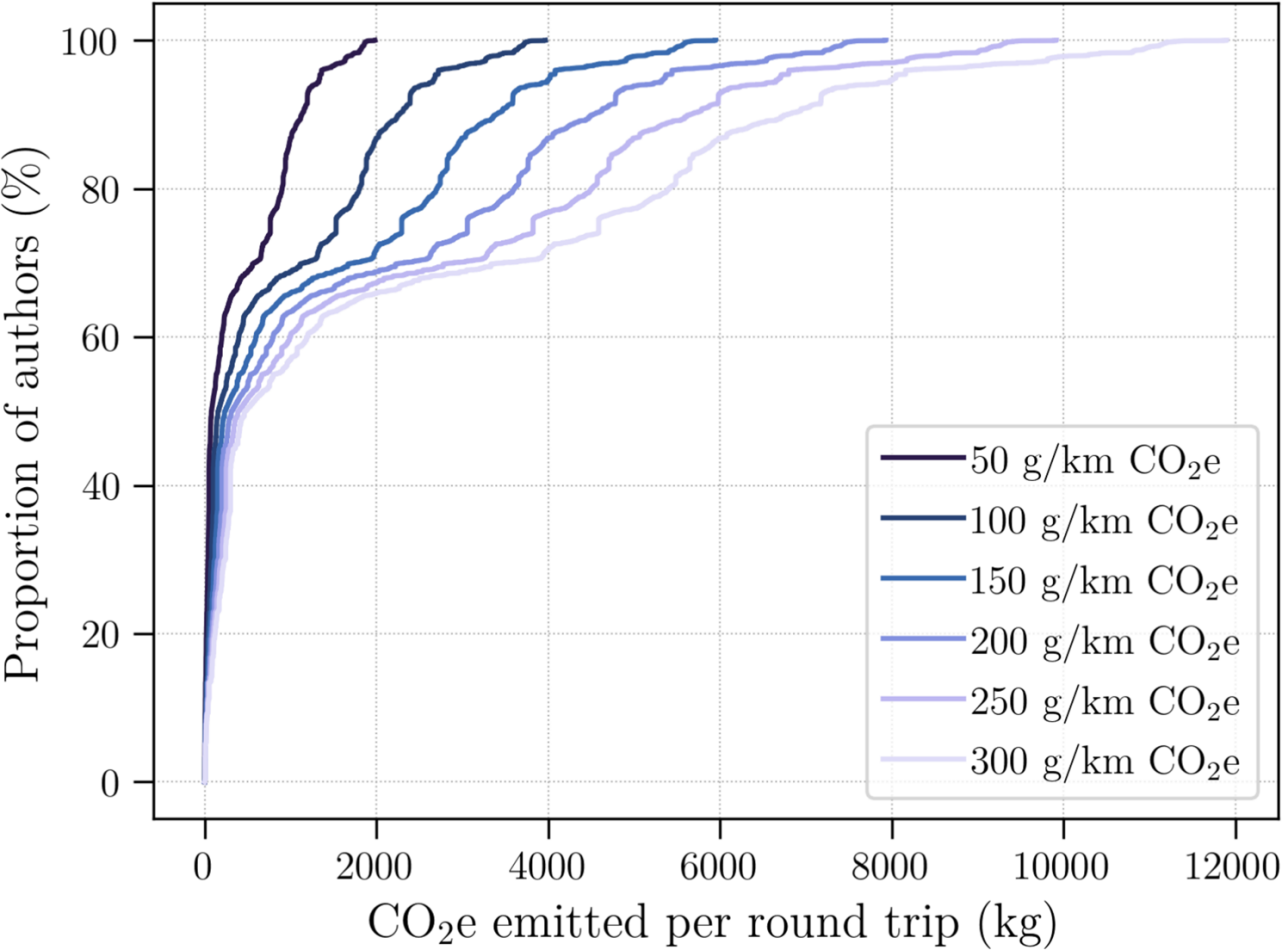
Cumulative distributions of the CO$_2$e emissions per author, for a round-trip to their studied volcano, for all authors ($n=8281$) having published in articles ($N=1582$) targetting the 25 volcanoes from Fig. 7

We computed the CO$_2$e emissions of a round-trip to the studied volcano, for every author ($n=8281$, from $N=1582$ articles) who studied one of the 25 volcanoes (Fig. 8). This results in distributions (one per conversion factor) of how much CO$_2$e would be emitted per author, if they travelled to their target volcano once. A major limitation of our dataset is that we do not know which articles required travel to the volcano (e.g. fieldwork), and which articles did not involve travel (e.g. remote sensing, laboratory experiments, numerical modelling, analysis of legacy samples). For articles which did involve travel to the volcanoes, we do not know which authors travelled to the volcano and how many times. Furthermore, we only consider the direct physical distance between affiliation address and the target volcano, which will most generally be underestimating the actual travel distance. Nonetheless, our analysis gives an order of magnitude estimate of the CO$_2$e emissions related to an authorship. Assuming that the travelling authors are uniformly sampled from the overall author population, the average emissions per author should be representative of their emissions, regardless of how many authors actually travelled. We postulate that the majority of travelling authors will use a combination of car and plane travel, likely resulting in a conversion factor in the range 150–200 g/km of CO$_2$e. The corresponding average emissions are 1137–1896 kg CO$_2$e per travelling author, for a single round trip. The subset of articles considered here ($N=1582$) has an average of 6.4 authors. Assuming that 2–4 authors travelled to the target volcano once, we obtain average values in the range 2275–6066 kg CO$_2$e emitted per article. These numbers only consider travel to and from the studied volcano, and omit any additional travels (e.g. to access laboratory facilities).

## Conclusions

Our initial question was as follows: how far are volcanologists from volcanoes? Using bibliometric data from the Scopus database, we found that 87% of authors who published in the four main English-speaking volcanology journals are located within 1000 km of a Holocene volcano and within 2000 km of a volcano that erupted in the last 50 years. Exploring the database in more detail revealed that volcanologists located nearer volcanoes are more likely to lead publications (i.e. be first author) with a larger number of co-authors. We also found that authors in further positions from the lead author are often nearer volcanoes, though this correlation was very sensitive to the chosen dataset. We examined the distance from authors to the specific volcanoes they study and observed various distinctive trends: from mostly studied locally (e.g. Campi Flegrei) to mostly studied from afar (e.g. Merapi). Our analyses also allowed an estimate of the order of magnitude of the carbon footprint of fieldwork, which is 1–2 ton of CO$_2$ equivalent emissions per travelling author.

We began this project out of sheer curiosity: are we further from volcanoes than most of our colleagues? Yes, we (Cornwall-based co-authors) are further than 75–85% of our colleagues (depending upon the database). We do not mean that only volcanologists located close to active volcanoes should study them. We believe that all scientists should be excited by volcanoes, regardless of how distant they are, and that an inclusive community should have opportunities to suit all. However, major issues in the inclusivity and recognition of local researchers, in particular from “low-income” countries (e.g. Indonesia, Nicaragua) have previously been reported, and are also reflected in our dataset.

### Supplementary information

Both volcano lists (Holocene and 1974–2024) and both databases (Author and Affiliation) are provided as Supplementary Material (.csv files). A list of the 25 most studied volcanoes in our database is also supplied, along with specific volcano distance analysis results for each of these. A project website is under construction, where readers can discover the nearest volcano to their institution: https://gilles.seropian.io/nearest_volcano.

## Supplementary Information

Below is the link to the electronic supplementary material.

Supplementary file 1 (zip 1378 KB)

## Data Availability

All the data is provided as electronic Supplementary Material.
